# The Effect of the Plant Stabilisation Method on the Composition and Antioxidant Properties of Elderflower (*Sambucus nigra* L.) Extract

**DOI:** 10.3390/molecules28052365

**Published:** 2023-03-04

**Authors:** Małgorzata Tabaszewska, Elżbieta Sikora

**Affiliations:** 1 Department of Plant Product Technology and Nutrition Hygiene, Faculty of Food Technology, University of Agriculture in Krakow, Al. Mickiewicza 21, 31-120 Krakow, Poland; 2 Department of Organic Chemistry and Technology, Faculty of Chemical Engineering and Technology, Cracow University of Technology, Warszawska 24, 31-155 Krakow, Poland

**Keywords:** elderflower extract, stabilisation method, antioxidant activity, lyophilisation, air drying, freezing

## Abstract

Elderflower extracts are known to be a source of valuable substances that show a wide spectrum of biological activity, including antibacterial and antiviral properties, which demonstrate a degree of effectiveness against SARS CoV-2. In this work, the influence of fresh inflorescence stabilisation methods (freezing, air drying, and lyophilisation) and extraction parameters on the composition and antioxidant properties of the extracts were studied. Wild elderflower plants growing in the Małopolska Region of Poland were studied. Antioxidant activities were evaluated by 2,2-diphenyl-1-picrylhydrazyl free radical-scavenging ability and ferric-reducing antioxidant power assays. The total phenolic content was determined using the Folin-Ciocalteu method and the phytochemical profile of the extracts was analysed using HPLC. The obtained results showed that the best method for the stabilisation of elderflower was lyophilisation, and the determined optimal maceration parameters were 60% methanol as a solvent and a process time of 1–2 days.

## 1. Introduction

In recent years, there has been a growing demand on the market for natural, plant based raw materials that are environmentally friendly and safe for humans. These raw materials can be used in food, supplements, and cosmetics [1]. Plants are a source of natural components, such as glycosides, alkaloids, and essential oils, which demonstrate various influence on physiological processes in human and animal organisms [2].

Among others, black elder (*Sambucus nigra* L.), also known as elderberry, has attracted attention due to its easy cultivation and high content of bioactive compounds. The plant belongs to the honeysuckle family (Caprifoliaceae). The *Sambucus* genus includes over 20 species of bushes and small trees that can be found in wet areas exposed to the sun in the majority of countries in Europe, Asia, Northern Africa, and North America [3,4]. Black elder can grow up to ten metres tall. It has an alternating arrangement of imparipinnate elliptical leaves that are 3–9 cm long. The bark is grey or brown-grey and the branches are grey or green-grey with a white core. The cream-coloured flowers, which are ca. 5 mm in diameter, have five stamens, and form a flat, umbel inflorescence with a diameter of 10–20 cm. The plant produces fleshy, dark purple or black berries with three to six seeds [2,5].

In the case of *Sambucus nigra* L., all parts of the plant (flowers, bark, leaves, and fruits) are rich sources of phytochemicals, although the elderflowers contain more bioactive compounds than the other parts of the plant [6]. Among other substances, there are flavonoids (mainly kaempferol, quercetin, isoquercetin, rutin, astragalin, and hyperoside), multiple phenolic acids (including caffeic, *p*-coumaric, ferulic, and chlorogenic acids) [7], organic acids, tannins, vitamins (B, C), and mineral salts. Moreover, the flowers also contain lipophilic active substances, e.g., triterpenes (mainly β-amyrin, α-amyrin, lupeol, cycloartenol, ursolic acid, etc.), as well as phytosterols (e.g., β-sitosterol, stigmasterol), and choline [8,9,10]. Additionally, there is 0.03–0.14% of essential oil in the elderflower [11]. Analysis of elderflowers revealed the presence of sixteen amino acids, including those that are most important for the human body: valine, threonine, methionine, isoleucine, leucine, lysine, histidine, and phenylalanine. The total content of amino acids in the flowers was 8.96% [12,13]. It should be emphasised that in contrast to bark, leaves, seeds and raw or unripe fruits, elderflowers do not contain the cyanogenic glycoside sambunigrin, which is potentially toxic because it can release cyanide [14].

The elderflower extracts are characterised by a flowery, pleasant odour and sweet, fruity, and honey-like taste, which make them a suitable aroma additive to many products [15,16]. Apart from their favourable sensory qualities, the extracts show a wide spectrum of nutritional and healing properties. They are characterised by their expectorant and diuretic properties, as well as their ability to strengthen the human immune system. Moreover, the extracts of elderflowers protect and strengthen the mucous membrane of the airways and show diaphoretic, purifying, cooling, and analgesic properties [17]. Additionally, extracts of *Sambucus nigra* L. inflorescences as well as the fruit extracts of the plant, exhibit a broad antiviral and anti-inflammatory effect which can be applied to fight against different types of viruses, including influenza and SARS CoV-2 [10,18,19,20,21,22].

Furthermore, elderflower extracts protect against strokes, alleviate headaches, and support sugar use in diabetes. When used externally, the extracts alleviate skin inflammation [23]. As a source of antioxidant active compounds, they can be applied to protect the human organism against the negative impact of free radicals [24,25].

For years, the black elderflower extracts have been used in natural medicine. Currently, the extracts are applied as components of dietary supplements [26] and as multifunctional raw materials of cosmetic products [27]. When used in cosmetic formulations, in addition to their antioxidant and moisturising effects on the skin, they influence the quality of the cosmetic products, enabling a reduction or even the elimination of synthetic preservatives [28].

Moreover, due to the rich content of bioactive substances, elderflower extracts are used as ingredients in regional food and beverages, such as teas, tinctures, and syrups [26].

It is generally known that the growth environment and methods of production significantly affect the composition and properties of the plant extracts [29,30,31,32]. In our work, the extracts obtained from the elderberry flower (*Sambucus nigra* L.) growing in the natural habitat in the Małopolska Region of Poland were prepared. The extraction procedures included maceration, a method convenient and very suitable for thermolabile plant material. The influence of stabilisation methods for fresh flowers and the conditions of the extraction process (extraction time, type, and concentration of the applied eluent) on the properties of the extracts were studied.

## 2. Results and Discussion

The elder inflorescences are raw materials that are rich in bioactive compounds. Unfortunately, they are seasonal raw materials, not durable, and only have a short harvest period. To maintain the availability of the active compounds throughout the whole year, the raw material of fresh plants have to be preserved (stabilised) during storage and before performing the extraction processes [32].

The influence of fresh flower stabilisation methods and the parameters of the extraction process on the composition and antioxidant properties of methanol and ethanol *Sambucus nigra* flowers extracts were studied. The total phenolic content was determined by the Folin-Ciocalteu method. The DPPH radical-scavenging ability and ferric-reducing antioxidant power assays were used to determine the antioxidant properties of the extracts. Table 1, Table 2, Table 3 and Table 4 present the obtained results.

The obtained results indicated that the method of stabilisation of the fresh plant material affects the quality of the obtained elderflower extracts.

The data presented in Table 1 shows that out of all the studied methods of preserving fresh elder inflorescences, the best one was the freeze-drying process (lyophilisation). When comparing the total content of polyphenols in the extract from the fresh, frozen, air-flow dried, and lyophilised plant material, it is clear that the highest concentration of the active compounds was obtained in the case of the freeze-dried raw materials. It was even higher than in the case of the extract obtained from fresh inflorescences. Our results are in accordance with those obtained through other researches, in which the following observations were made: an increase of polyphenol content and antioxidant capacity of extracts obtained from freeze-dried raw material, and an increase in the content of total polyphenols in freeze-dried material in the cases of blueberry (19–25%), raspberry (19–34%) [33], jujube (36%) [34], and papaya (to 3.2-fold) [35]. In the case of papaya, there were also increases indicated in the total flavonoid content (to 200%). Moreover, increases of antioxidant activity against the ABTS radical were observed for lyophilised blueberry (1–27%), raspberry (13–82%) [33], and guabiju (38%) [36]. By contrast, Shofian et al. [37] observed an increase in the ability of freeze-dried muskmelon and papaya to reduce iron ions (FRAP) of 40% and 80%, respectively [38].

Despite the above-mentioned, this is not a universal rule, as similar changes (the increase in the total polyphenol content) have been observed in the case of dried fruits obtained by air blowing. With regard to dried fruits obtained in such a manner, the polyphenol levels were 6–9% in apple [39], 80% in blueberry and raspberry [33], a 6 to 10-fold increase in fig [40], and 176% in papaya [35]. Moreover, in the case of raw materials obtained by the air blowing method, an increase in antioxidant activity was also observed in the dried materials obtained from blueberry (43%), unblanched raspberry (33%), blanched raspberry (43%) [33], guabiju (27%), red guava (66%) [36], and figs (two-fold) [40]. The air-dried figs also showed activity with regard to the inhibition of ABTS and DPPH radicals [38].

When discussing the influence of extraction process parameters on the quality of the fresh elderberry inflorescence extracts, it should be emphasised that the extraction time affects the properties of the macerates, but only during the first twenty-four hours of the process (Table 2).

The largest amounts of flavonoids and polyphenolic compounds, the important active neutralising free radicals, were obtained after the first day of the maceration process. An extension on the extraction time had no significant effect on the content of polyphenols in the extracts. The higher amount of the active compounds was extracted using methanol as an extracting agent (Table 3).

The alcohol concentration in the eluent solution influences the efficiency of the extraction process. The greatest antioxidant properties assessed by the FRAP method, and the total flavonoid and polyphenol content in the extract was found in the case of 60% solvent (Table 4).

The greatest scavenging capacity on the DPPH free radical was found in the case of the extract obtained with the use of a 70% alcohol solution.

The scavenging capacity on the DPPH free radical of the fresh inflorescences was on a similar level to that obtained in the research by Młynarczyk and Walkowiak-Tomczak [41], who studied the content of the bioactive compounds in 80% methanol extracts from the inflorescences of different elder varieties taken from several natural sites. Their research shows that the scavenging capacity on the DPPH free radical and the extraction efficiency of the total polyphenol content was lower after air-flow drying, in contrast to the performed study. However, the total polyphenol content was found to be at a similar level as that presented by Młynarczyk et al. [42]. In the case of water extracts from the elder inflorescences which were obtained through microwave extraction, FRAP in the range of 29–42 mMFe^2+^/100 g was obtained [43]. Slightly higher FRAP values were obtained in this study most likely because a different extraction method and extracting agents were used. The scavenging capacity on the DPPH free radical increased with the extraction time. The most important increase (ca. 60%) was observed between the first and second day of the self-extraction. Research by Diankov and Parlapanska [44] showed that for up to 90 min, the scavenging capacity on the DPPH free radical of the elder inflorescence extract initially increased and then stabilised, which is similar to what was found in this study (1–3 extraction days). As observed by Diankov and Parlapanska [44], similarly in this study, the amount of extracted total polyphenols and the dry extract increased with the extraction time (120 min), since the start of the experiment to its second day.

It should be emphasised that although methanol extracts were characterised by a better content of bioactive compounds and antioxidant activity, taking into account the potential application of elderflower extracts (in the food or cosmetics industries where ethanol extracts are preferred), the next stage of the study involved carrying out a detailed analysis of the profiles of polyphenolic compounds in the extract, for the optimal ethanol extract obtained using freeze-dried elderberry inflorescences.

**Figure 1:**
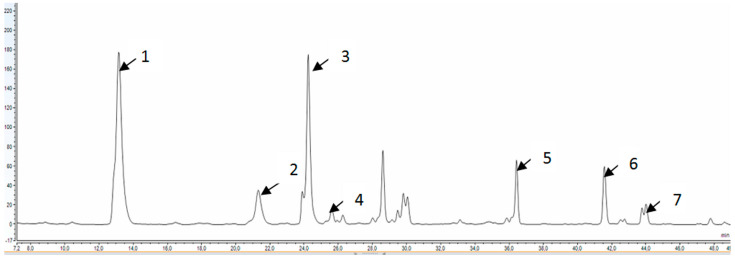
HPLC chromatogram of polyphenolic compounds in the ethanol extract of elder inflorescences (Peaks: 1—caffeic acid; 2—*p*-coumaric acid; 3—rutin; 4—ferulic acid; 5—chlorogenic acid; 6—*t*-cinnamic acid; and 7—kaempferol).

Figure 1 and Table 5 present the polyphenolic profile of the ethanol extract of elder inflorescences, analysed using HPLC. Mostly, rutin, kaempferol, and caffeic acid were identified among polyphenol compounds found in ethanol macerate from the freeze-dried elder inflorescence ethanol (Table 5). These results are similar to those obtained by Ferreira et al. [13], who identified these phenolic compounds in extracts of elderberry inflorescences [13].

In the study by Młynarczyk et al. [42], the results of the polyphenol profile determination using the HPLC-MS method were presented. Among others, the following polyphenols were identified in the elder inflorescence: chlorogenic, *p*-coumaric, caffeic acids, kaempferol (identified also in this study), quercetin, catechin, and epicatechin, among others. In the work of Sidor and Gramaza-Michałowska [9], myricetin and isorhamnetin, among others compounds, were also determined in the elder inflorescences.

Referring to the influence of the stabilisation method on the composition of the extract, the result of our studies showed that the extracts obtained from freeze-dried inflorescences contained a total content of polyphenols that was over 60% more than in the case of the extracts obtained from the air-flow dried inflorescences. A similar result, with regard to the high total content of polyphenol in the ethanol extracts prepared from daylily flowers, was obtained by Mao et al. [45]. Also, in a study performed by Chen and coworkers [46], the freeze-drying stabilisation methods of fresh plant material (*E. purpurea* flowers) produced extracts that were richer in polyphenol compounds [46].

## 3. Materials and Methods

### 3.1. Plant Material

The inflorescences of elder (*Smabucus nigra* L.) were collected on a sunny day, in a natural site in Lesser Poland Voivodeship (49.8167° N, 20.5667° E), Poland. The plants were identified as *Sambucus nigra* L. by the morphological comparison of leaves, flowers, tree bark, and berries according to Rutkowski [47]. The collected fresh inflorescences were selected; fragments of plants that were unsuitable for processing were removed; and then the high-quality plant material was spread on filter paper for fourty-five minutes at a room temperature of 21–22 °C.

### 3.2. Methods of Elderflower Stabilisation

Next, the prepared flowers were divided into four samples in order to perform the stabilisation process of the fresh flower, using various methods. In the case of the first sample, the fresh flowers were directly analysed without pretreatment. The second sample of the flower material was frozen at T = −40 °C (single-door freezer, RoHS, Poland) and stored in the freezer until the analysis. The third sample was dried in an air-flow dryer at 35 °C, with forced-air circulation (SLW 115 STD air dryer, Pol-Eko-Aparatura Sp. J., Poland). The fourth elderflower sample was frozen at −40 °C and then freeze-dried using a freeze dryer (Gamma 1–16 LSC, Christ, Germany). In this case, the freeze-dried process consisted of two stages: first, the initial drying process was performed (the temperature of the raw material was −30 °C, the temperature of the condenser was −52 °C, and the shelf temperature was kept at +20 °C), and the further drying step was then conducted for approximately six hours at a shelf temperature of +30 °C.

### 3.3. Parameters of the Elderflower Extraction

Maceration was chosen as the extraction method. All comparative samples were macerated in water solutions of ethanol or methanol. Water solutions of the alcohol were used as the eluents at concentrations of 50, 60, 70, and 90% (*v*/*v*). The weighed stabilised flowers were homogenised with the alcohol solution of adequate concentrations for 90 s, with a mixing speed of 12,000× *g*, using a DI 25 basic homogeniser (Ika Warke, Düsseldorf, Germany). In all cases, the ratio of fresh plant material to eluent were 1:125. Next, after the specified period of time, the inflorescences were separated from the liquid extract using a paper filter (medium thickness). The first sample (“0”) was separated and analysed directly after the homogenisation process was stopped. The rest of the extract samples were covered with a parafilm and stored in the refrigeration cabinet at temperature T = 4 °C, for self-extraction for one, two, and three days.

### 3.4. Analysis of the Properties of the Elderflower Extracts

The phytochemical profiles of the elderflower extracts were analysed by chromatographic methods, using HPLC Dionex UltiMate 3000 system which consisted of a DAD Thermo Scientific detector (Germering, Germany). The antioxidant properties of the extracts were evaluated using a DPPH radical scavenging capacity assay, and the FRAP method. The total phenolic content was determined by the Folin-Ciocalteu method. In all cases, the spectrophotometric analysis was performed using HITACHI U-2900 UV-Vis spectrophotometer (Hitachi, Europe Ltd., Stoke Poges, UK).

#### 3.4.1. Ferric-Reducing Antioxidant Power (FRAP) Assay

Ferric-ion-reducing antioxidant power (FRAP) was determined with a spectrophotometric method [48]. A mixture of a standard solution of 10 mmol/L TPTZ (ferrum-2,4,6-tripyridyl-S-triazine) solution in 40 mmol/L HCl: 20 mmol ferric chloride: 300 mmol sodium acetate buffer (pH 3.6) at an amount of 1:1:10 and an appropriate amount of the extract was vortexed using a Labnet vortexer (270× *g*; 20 s; Edison, NJ, USA), and subsequently placed in an incubator (T = 37 °C) (Heraeus instrument T12, Hanau, Germany) for 10 min. It was then centrifuged with an MPW-260R centrifuge (Warsaw, Poland) for 2 min (1600× *g*) and the absorbance of the separate solution was measured at a wavelength of 595 nm and compared to the blank test. The value of antioxidant activity was read from a calibration curve and expressed in µmol Fe^2+^.

#### 3.4.2. DPPH Radical Scavenging Capacity Assay

Antioxidant activity was also determined with a spectrophotometric method using DPPH free radical (1,1-diphenyl-2-picrylhydrazyl), as described by Brand-Williams et al. [49]. Briefly, 1 mL of elderflower extract and 3 mL 0.06 mmol/L ethanol DPPH solution were vigorously mixed and incubated for 10 min at room temperature. The absorbance of the mixtures was measured at a wavelength of 516 nm. The antioxidant activity was expressed in μmol of Trolox [49].

#### 3.4.3. Total Flavonoid Content Determination

The total flavonoid content was determined according to the method described by Ardestani et al. [50,51]. The mixture of the extract consisting of 2 mL 15% (*w*/*v*) sodium nitrite (0.15 mL), 10% (*w*/*v*) aluminium chloride (0.15 mL), 4% (*w*/*v*) sodium hydroxide (2 mL), and 0.2 mL distilled water, was vortexed using a Labnet vortex mixer (270× *g*; 20 s; Edison, NJ, USA) and stored in a dark place for 15 min (21–22 °C; room temperatures). After the incubation time had elapsed, the absorbance of the samples was measured at a wavelength of 510 nm. The total flavonoids content was expressed as (+)- catechin concentration.

#### 3.4.4. Total Polyphenol Content Determination

The total polyphenol content was determined by the Folin-Ciocalteau method [52]. The Folin-Ciocalteau reagent (0.125 mL) and 25% (*w*/*v*) sodium carbonate (0.25 mL) was added to an adequate amount of the extract (estimated to obtain a 0.3–0.8 range of the absorbance reading), and then the whole solution was mixed using the vortex mixer (Edison, NJ, USA). The samples were left in a dark place for 60 min (at room temperature). The absorbance was then measured at a wavelength of 675 nm using the UV-vis spectrophotometer. The total polyphenol content was read from the calibration curve for (+)- catechin.

#### 3.4.5. HPLC Analysis of the Polyphenols Profile

The samples for the HPLC polyphenols determination were prepared according to the modified method of Klimczak et al. [53]. Sodium hydroxide (2 mol) at an amount of 1:1 (*v*/*v*) was added to the extract which was obtained from the lyophilised inflorescences according to the description above for the ethanol extracts with the “0” extraction time. The sample was mixed with the help of a Labnet vortex mixer (Edison, NJ, USA) and left in a dark place for 4 h (21–22 °C; room temperature). The hydrochloric acid water solution (2 mol) was added to the samples in order to obtain the pH value of 2.1–2.6 (Metrohm pH-meter, Herisau, Switzerland) and moved to a volumetric flask with 1% (*w*/*v*) L-ascorbic acid dissolved in HPLC-grade methanol. Before the chromatographic analysis, the samples were centrifuged (18,000× *g*; 20 min; 4 °C) with an MPW—260R centrifuge (Warsaw, Poland), and filtered through a PTFE-L filter with a 22 µm mesh. The samples were then kept at 4 °C before they were dosed into the HPLC column. The XBridge C18 250 × 4.6 mm, 3.5 µm, column together with the XBridge TM C18 20 × 4.6, 3.5 µm pre-column by Waters (Wexford, Ireland) were used. The mobile phase consisted of two eluents: A—2% (*v*/*v*) water solution of acetic acid; and B—100% acetonitrile. The eluents’ flow rate was 0.8 mL ·min^−1^. The chromatographic analysis lasted for 80 min: Eluent A—15 min, 14%; 20 min, 18%; 30 min, 25%; 55 min, 55%; and 62 min, 100%.

### 3.5. Dry Extract Content Determination

The content of the dry extracts was determined according to the method described by Tabaszewska [27]. The elderflower alcohol extracts were condensed using a 4000 Heidolph laboratory vacuum evaporator (Schwabach, Germany) with a water bath at 40 °C, and at 22 rpm of the flask with the extract. The remaining part of the liquid was freeze-dried (Gamma 1–16 LSC freeze dryer, Christ, Germany) and the dry extracts were then weighed.

### 3.6. Statistical Analysis

A multiple factor analysis of variance was performed using the STATISTICA 13.1 software (TIBCO Software Inc., Palo Alto, CA, USA). All of the analyses were performed in three repetitions (*n* = 3) and were expressed as a mean average ± standard error. The significance between the mean values was determined using a post-hoc Tukey test at *p* < 0.05.

## 4. Conclusions

The elder inflorescences are rich sources of numerous active compounds and show a wide spectrum of nutritional and healing properties, including strong antioxidant activity. Several extracting methods were used to obtain elderflower extracts. Maceration is one of the most convenient methods that are especially suitable for thermolabile plant material, such as elder inflorescences. The flowers are seasonal raw material, and therefore, to maintain availability of the active compounds during storage throughout the whole year, the fresh plant needs to be preserved (stabilised). Both the stabilisation methods and extraction process parameters influence the quality of the extract.

The obtained results showed that the best method for preserving the fresh elder inflorescences is the lyophilisation process. The comparison of the extracts from the fresh, frozen, air-flow drying, and lyophilised plants indicated that the highest concentration of polyphenols, and consequently the highest antioxidant efficiency, were obtained in the case of the freeze-dried raw material. The elderflower extract obtained from lyophilised plant material contained from 96% to over 500% more polyphenols when compared with those derived from other methods of raw-material stabilisation. In addition, the same extract is characterised by over 50% to 90% higher ferric-ion reduction capacity and over five times higher antioxidant activity against the DPPH radical, compared with the extract from elderflower stabilised by freezing or using the air-drying method. The optimal extraction was performed with 60% methanol, for 1–2 days, by self-extraction from the inflorescences preserved with the freeze-drying method. However, considering the potential applications of elderflower extracts (in the food and cosmetics industries), ethanol extracts are preferred.

## 5. Patents

Tabaszewska, M. Method for obtaining lyophilised plant extract as the active component of products, preferably cosmetic products. 2021, Patent No. 240152.

## Figures and Tables

**Table 1:** The influence of the stabilisation methods of raw material on the antioxidant properties of the elderflower extracts (FRAP, DPPH), the total contents of flavonoids and polyphenols, and the content of the dry extract in grams (SE ±, *n* = 96).

	FRAP [µmol Fe^2+^]	DPPH [µmol TE]	Flavonoids [mg(+)Catechin]	Total Polyphenols [mg(+)Catechin]	Dry Extract [g]
Stabilisation method					
**Fresh**	473.60 ± 14.9 ^b^	567.96 ± 17.0 ^a^	511.58 ± 23.2 ^b^	2072.50 ± 76.1 ^b^	0.257 ± 0.005 ^d^
**Frozen**	77.40 ± 2.5 ^a^	243.55 ± 29.8 ^a^	81.40 ± 4.0 ^a^	619.04 ± 22.9 ^a^	0.189 ± 0.004 ^b^
**Air-flow drying**	685.59 ± 45.1 ^c^	1029.43 ± 221.8 ^b^	767.56 ± 41.2 ^d^	2813.32 ± 179.7 ^c^	0.234 ± 0.014 ^c^
**Freeze-drying**	777.67 ± 30.1 ^d^	5131.51 ± 505.9 ^c^	575.16 ± 25.4 ^c^	4070.35 ± 106.5 ^d^	0.185 ± 0.004 ^a^

The data are the mean value of the smallest square. The same letters in the column indicate no significant statistical differences at *p* < 0.05; TE—Trolox equivalents.

**Table 2:** The influence of the extraction time on the antioxidant properties of the elderflower extracts (FRAP, DPPH), the total contents of flavonoids and polyphenols, and the content of the dry extract in grams (SE ±, *n* = 96).

	FRAP [µmol Fe^2+^]	DPPH [µmol TE]	Flavonoids [mg(+)Catechin]	Total Polyphenols [mg(+)Catechin]	Dry Extract [g]
Extraction time [days]					
**0**	283.84 ± 28.1 ^a^	1909.31 ± 400.7 ^a^	298.70 ± 17.5 ^a^	1598.03 ± 163.0 ^a^	0.141 ± 0.009 ^a^
**1**	632.77 ± 47.9 ^d^	3050.42 ± 541.5 ^b^	559.39 ± 41.74 ^b^	2611.04 ± 176.1 ^b^	0.240 ± 0.006 ^b^
**2**	586.42 ± 43.3 ^c^	3446.94 ± 664.4 ^b^	548.79 ± 39.34 ^b^	2752.89 ± 182.4 ^c^	0.244 ± 0.007 ^c^
**3**	511.21 ± 40.9 ^b^	3481.96 ± 672.8 ^b^	539.48 ± 47.0 ^b^	2613.27 ± 211.1 ^b^	0.240 ± 0.007 ^b^

The data are the mean value of the smallest square. The same letters in the column indicate no significant statistical differences at *p* < 0.05; TE—Trolox equivalents.

**Table 3:** The influence of the kind of solvent used on the antioxidant properties of the elderflower extracts (FRAP, DPPH), the total contents of flavonoids and polyphenols, and the contents of the dry extract in grams (SE ±, *n* = 192).

	FRAP [µmol Fe^2+^]	DPPH [µmol TE]	Flavonoids [mg(+)Catechin]	Total Polyphenols [mg(+)Catechin]	Dry Extract [g]
Extracting agent (solvent) type					
**Ethanol**	479.86 ± 29.2 ^a^	2747.51 ± 382.9 ^a^	482.38 ± 28.6 ^a^	2347.58 ± 130.4 ^a^	0.210 ± 0.007 ^a^
**Methanol**	527.26 ± 32.7 ^b^	3157.79 ± 432.4 ^b^	488.08 ± 27.9 ^a^	2440.02 ± 141.2 ^b^	0.222 ± 0.006 ^b^

The data are the mean value of the smallest square. The same letters in the column indicate no significant statistical differences at *p* < 0.05; TE—Trolox equivalents.

**Table 4:** The influence of the concentration of the solvent used on the antioxidant properties of the elderflower extracts (FRAP, DPPH), the total contents flavonoids and polyphenols, and content of the dry extract in grams (SE ±, *n* = 96).

	FRAP [µmol Fe^2+^]	DPPH [µmol TE]	Flavonoids [mg(+)Catechin]	Total Polyphenols [mg(+)Catechin]	Dry Extract [g]
Extracting agent concentration [%]					
**50**	451.00 ± 38.4 ^a^	2372.29 ± 460.9 ^a^	463.88 ± 38.6 ^a^	2353.21 ± 180.6 ^b^	0.211 ± 0.009 ^a^
**60**	532.13 ± 44.5 ^c^	3274.97 ± 605.6 ^b^	530.02 ± 43.7 ^b^	2565.85 ± 192.9 ^d^	0.220 ± 0.009 ^b^
**70**	526.87 ± 47.0 ^c^	3454.42 ± 656.5 ^b^	485.67 ± 40.9 ^a^	2433.06 ± 211.1 ^c^	0.223 ± 0.009 ^b^
**90**	504.25 ± 45.2 ^b^	2721.55 ± 575.1 ^a^	461.83 ± 36.5 ^a^	2223.10 ± 183.2 ^a^	0.211 ± 0.009 ^a^

The data are the mean value of the smallest square. The same letters in the column indicate no significant statistical differences at *p* < 0.05; TE—Trolox equivalents.

**Table 5:** Content of the polyphenols identified in the 70% ethanol extract obtained from freeze-dried elder inflorescences (SE ±, *n* = 4).

Name of Active Component	mg in 100 mL
Caffeic acid	10.96 ± 0.008
*p*-coumaric acid	1.82 ± 0.003
Rutin	38.32 ± 0.097
*t*-cinnamic acid	0.67 ± 0.002
Kaempferol	14.30 ± 0.020
Chlorogenic acid	5.14 ± 0.015
Ferulic acid	0.59 ± 0.000

## Data Availability

The data presented in this study are available on request from the corresponding author.

## Sample Availability

Samples of the compounds are not available from the authors.
